# Chicken-eaters and pork-eaters have different gut microbiota and tryptophan metabolites

**DOI:** 10.1038/s41598-021-91429-3

**Published:** 2021-06-07

**Authors:** Jie Shi, Di Zhao, Fan Zhao, Chong Wang, Galia Zamaratskaia, Chunbao Li

**Affiliations:** 1 Key Laboratory of Meat Processing and Quality Control, MOE, Key Laboratory of Meat Processing, MARA, Jiangsu Synergistic Innovation Center of Meat Processing and Quality Control, College of Food Science and Technology, Nanjing Agricultural University, Nanjing, 210095 People’s Republic of China; 2 Department of Molecular Sciences, Swedish University of Agricultural Sciences, 75007 Uppsala, Sweden; 3 South Bohemian Research Center of Aquaculture and Biodiversity of Hydrocenoses, Faculty of Fisheries and Protection of Waters, University of South Bohemia in Ceske Budejovice, Zatisi 728/II, 389 25 Vodnany, Czech Republic

**Keywords:** Biochemistry, Microbiology

## Abstract

This study was aimed to evaluate the differences in the composition of gut microbiota, tryptophan metabolites and short-chain fatty acids in feces between volunteers who frequently ate chicken and who frequently ate pork. Twenty male chicken-eaters and 20 male pork-eaters of 18 and 30 years old were recruited to collect feces samples for analyses of gut microbiota composition, short-chain fatty acids and tryptophan metabolites. Chicken-eaters had more diverse gut microbiota and higher abundance of *Prevotella 9, Dialister, Faecalibacterium, Megamonas,* and *Prevotella 2*. However, pork-eaters had higher relative abundance of *Bacteroides, Faecalibacterium, Roseburia, Dialister,* and *Ruminococcus 2*. In addition, chicken-eaters had high contents of skatole and indole in feces than pork-eaters, as well as higher contents of total short chain fatty acids, in particular for acetic acid, propionic acid, and branched chain fatty acids. The Spearman’s correlation analysis revealed that the abundance of *Prevotella 2* and *Prevotella 9* was positively correlated with levels of fecal skatole, indole and short-chain fatty acids. Thus, intake of chicken diet may increase the risk of skatole- and indole-induced diseases by altering gut microbiota.

## Introduction

In recent decades, eating habits have changed dramatically^1^. These changes have various effects on human health^2^. Several chronic diseases, such as cardiovascular diseases and diabetes are closely related to long-term unhealthy dietary habits^3^. In many countries, meat is an indispensable part of routine meals because meat is rich in high-quality protein, vitamins and minerals^4,5^. Preferences for different types of meat vary between and within countries depending on demographic and cultural influences. Protein composition differs between different types of meat, which may modulate the composition of gut microbiota and formation of their metabolites^6^.

Meat and meat products are the most important source for L-tryptophan (L-trp), which can be transformed into skatole and indole by the gut microbiota in the large intestine. Skatole was shown to be pneumotoxic to humans and associated with hepatic encephalopathy and saccharo-butyric putrefaction, whereas increased indole and oxindole were observed in cirrhotic patients’ blood^7–10^. Dietary fiber and peptides can be degraded by the gut microbiota into short-chain fatty acids (SCFAs). SCFAs may affect lipid metabolism, bacterial fermentation and the capacity of intestinal absorption including the absorption rate of skatole in gut^11,12^.

Many studies have been focused on the effects of meat consumption on the composition of gut microbiota, however, only few studies investigated an impact of meat consumption on tryptophan metabolites. In a previous study, we found that a high fat high chicken or pork protein diet increased the abundance of gut microbiota associated with skatole and indole production in Wistar rats^13^. In the present study, we investigated the differences in composition of gut microbiota, tryptophan metabolites and SCFAs in feces in volunteers who frequently consume chicken or pork products.

## Results

### Skatole, indole and SCFAs in fecal samples

**Figure 1:**
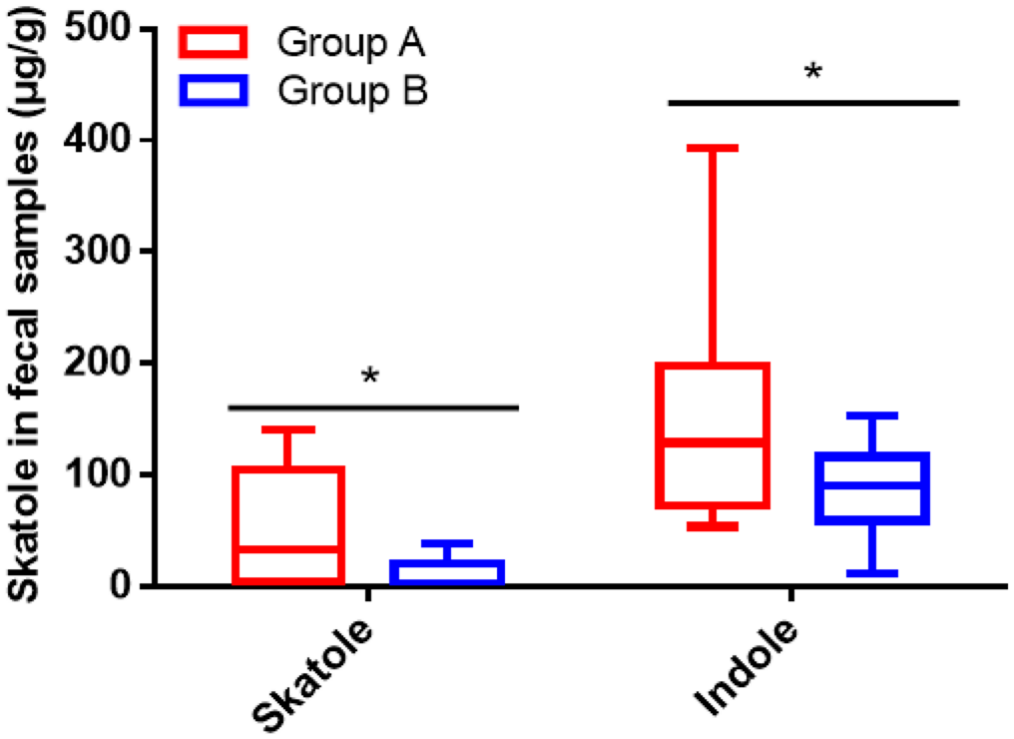
Chicken-eaters had higher levels of skatole and indole in feces than pork-eaters. The means were compared by Student’s t test. The asterisk (*) indicates significant difference between two groups (*P* < 0.05).

The concentrations of fecal skatole and indole were higher in chicken-eaters (*P* < 0.05, Fig. 1). Chicken-eaters also had significantly higher concentrations of total SCFAs as well as acetic acid, propionic acid, isovaleric acid, and branched chain fatty acids (BCFAs) than pork eaters (*P* < 0.05, Table 1).

**Table 1:** SCFAs levels of human fecal samples (μmol/g).

	Chicken-eaters	Pork-eaters
Acetic acid	41.3 ± 11.4^a^	25.7 ± 18.0^b^
Propionic acid	19.5 ± 8.8^a^	11.9 ± 2.9^b^
Isobutyric acid	0.7 ± 0.4	0.6 ± 0.4
Butyric acid	9.2 ± 3.3	10.3 ± 3.4
Isovaleric acid	1.2 ± 0.8	0.7 ± 0.5
Valeric acid	1.9 ± 1.1	1.3 ± 0.8
T-SCFAs	74.7 ± 23.0^a^	46.8 ± 22.6^b^
BCFAs	1.9 ± 1.2^a^	1.1 ± 0.9^b^

^a,b^Means differed significantly (*P* < 0.05).

### Gut microbiota composition and its correlations with skatole, indole and SCFAs

#### Richness and diversity

**Figure 2:**
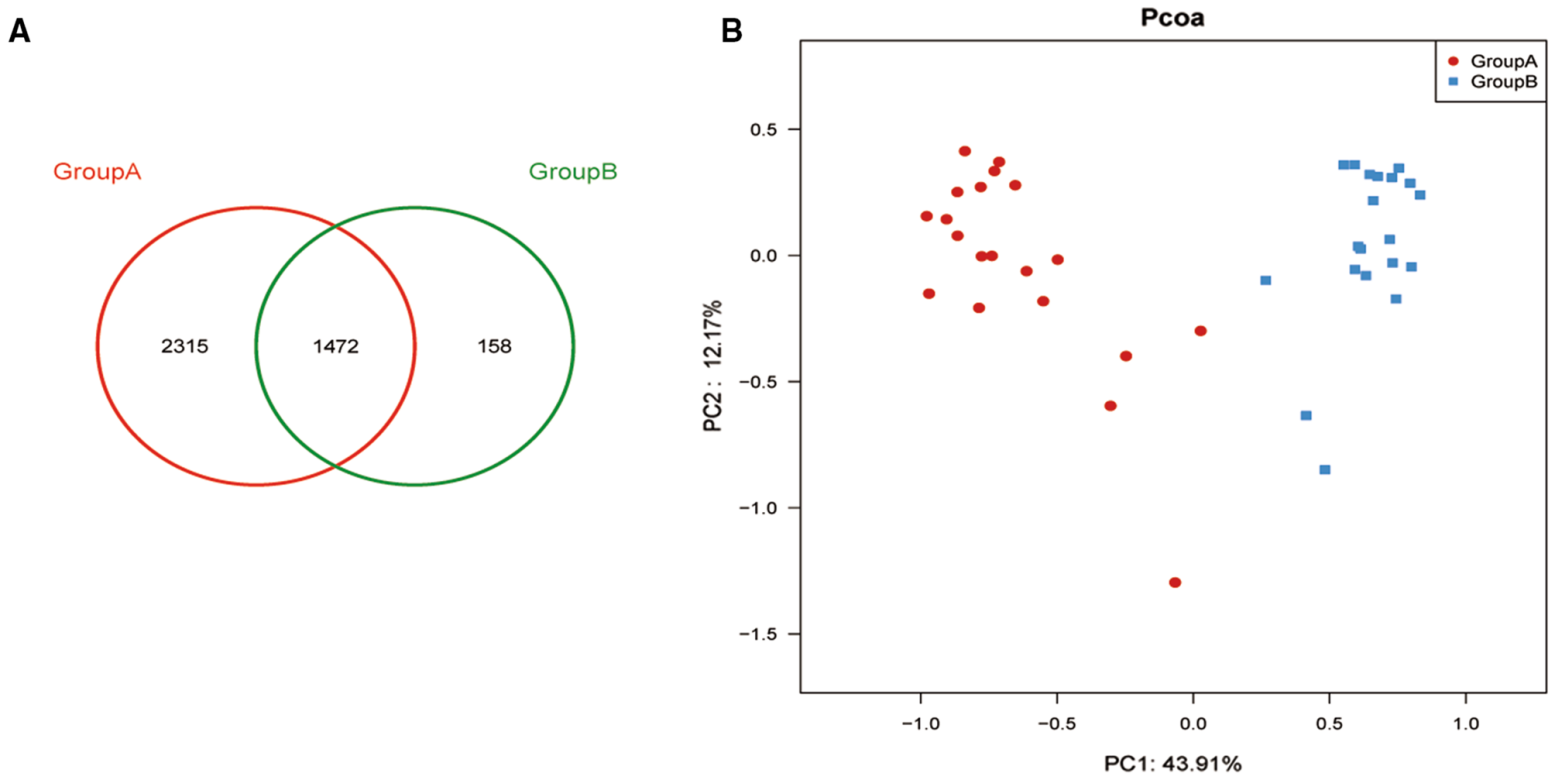
Chicken-eaters had higher diversity of the gut microbiota than pork-eaters. (**A**) Venn diagram of fecal microbiota at the OTU level. (**B**) PCoA plot of fecal microbiota at the OTU level. Each point represents one sample.

A total of 3787 and 1630 OTUs were identified in the fecal samples from chicken and pork eaters, respectively. The number of shared OTUs between the two groups was 1472. The numbers of OTUs specific for chicken- and pork-eaters were 2315 and 158, respectively (Fig. 2A). The diversity of gut microbiota in pork-eaters was relatively low, indicating that pork consumption reduced the diversity of gut microbiota. PCoA reveals responses of volunteers’ gut microbiota to the two dietary modes (Fig. 2B). The first and second principal components (PC1 and PC2) explained 56.1% of the total variance in gut microbiota composition, in which PC1 accounted for 43.9% and PC2 accounted for 12.2%. PC1 mainly explained the inter-group variation due to dietary effects, while PC2 explained the intra-group variation from individual volunteers. The two groups can be well separated in the first principal component, indicating that the gut microbiota responded differently to the two dietary modes. Analysis of the α diversity of samples in the two groups revealed that both Chao and Shannon indexes of pork-eaters were significantly lower than those in chicken-eaters (*P* < 0.01, Table 2), suggesting that pork protein reduced the diversity of gut microbiota.

**Table 2:** Alpha diversity of human fecal microbiota.

	Chicken-eaters	Pork-eaters
Chao	1945.7 ± 1917.6^a^	737.7 ± 119.0^b^
Shannon	4.6 ± 0.7^a^	4.0 ± 0.4^b^
Simpson	0.05 ± 0.05	0.05 ± 0.03

^a,b^Means differed significantly (*P* < 0.05).

#### Gut microbiota composition

**Figure 3:**
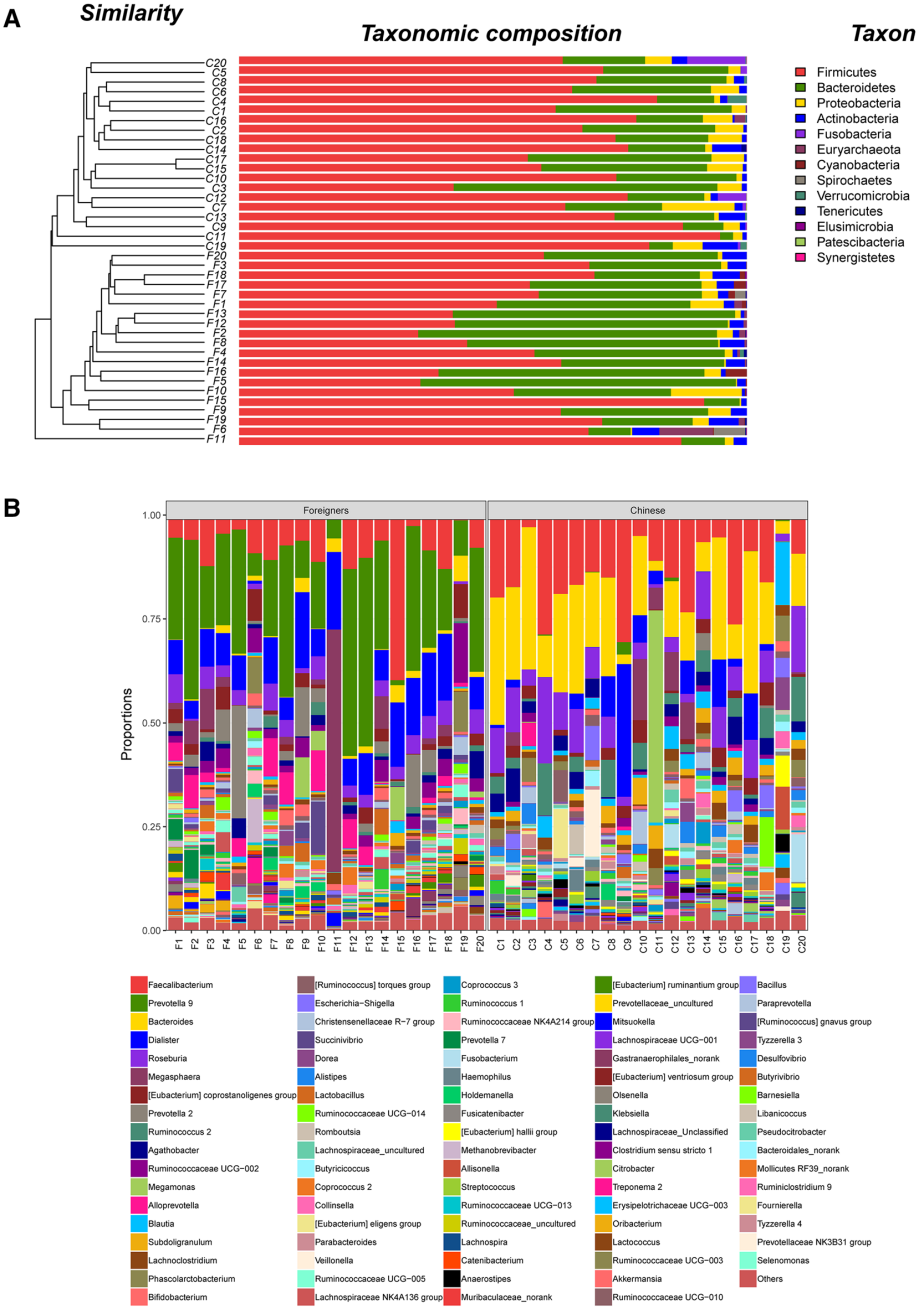
Composition of fecal microbiota at the phylum and genus levels. (**A**) Hierarchical clustering of fecal microbiota at the phylum level. Each line and bar represents one sample. (**B**) Composition of fecal microbiota at the genus level. Each column represents one sample and each color represents one genus.

At the phylum level, *Firmicutes*, *Bacteroidetes*, *Proteobacteria* and *Actinobacteria* were the four most abundant phyla, with the range of 35.3–94.7%, 2.6–62.1%, 0.2–14.3% and 0.1–6.9%, respectively (Fig. 3A). In pork-eaters, *Firmicutes* had the highest relative abundance (58.2%), followed by *Bacteroidetes* (34.4%), *Actinobacteria* (2.8%) and *Proteobacteria* (2.6%). The dominant phyla in chicken-eaters were *Firmicutes* (71.2%), *Bacteroidetes* (21.3%), *Proteobacteria* (4.2%) and *Actinobacteria* (1.8%). Relative abundance of *Firmicutes* in pork-eaters was significantly higher than that in chicken-eaters (*P* < 0.01), but relative abundance of *Bacteroidetes* was lower in pork-eaters (*P* < 0.01).

At the genus level, the dominant bacteria are quite different (Fig. 3B). In chicken-eaters, relative abundance of *Prevotella 9* was the highest (22.45%), followed by *Dialister* (9.5%), *Faecalibacterium* (9.0%), *Megamonas* (4.6%), *Prevotella 2* (4.2%), *Roseburia* (3.6%), *Alloprevotella* (3.2%), *Ruminococcaceae UCG-002* (3.2%), *[Eubacterium] coprostanoligenes group* (2.5%), and *Succinivibrio* (1.7%). In pork-eaters, relative abundance of *Bacteroides* was the highest (17.3%), followed by *Faecalibacterium* (14.6%), *Roseburia* (7.4%), *Dialister* (4.6%), *Ruminococcus 2* (3.7%), *Blautia* (2.6%), *Megamonas* (2.6%), *Agathobacter* (2.5%), *Subdoligranulum* (2.1%), and *[Eubacterium] coprostanoligenes group* (2.1%). Interestingly, relative abundance of *Olsenella* was higher in chicken-eaters (0.5%) than in pork-eaters (0.03%). *Olsenella scatoligenes* has been reported a skatole-producing bacterium in the intestine of pigs^14^.

#### Linear discriminant analysis of fecal microbiota

**Figure 4:**
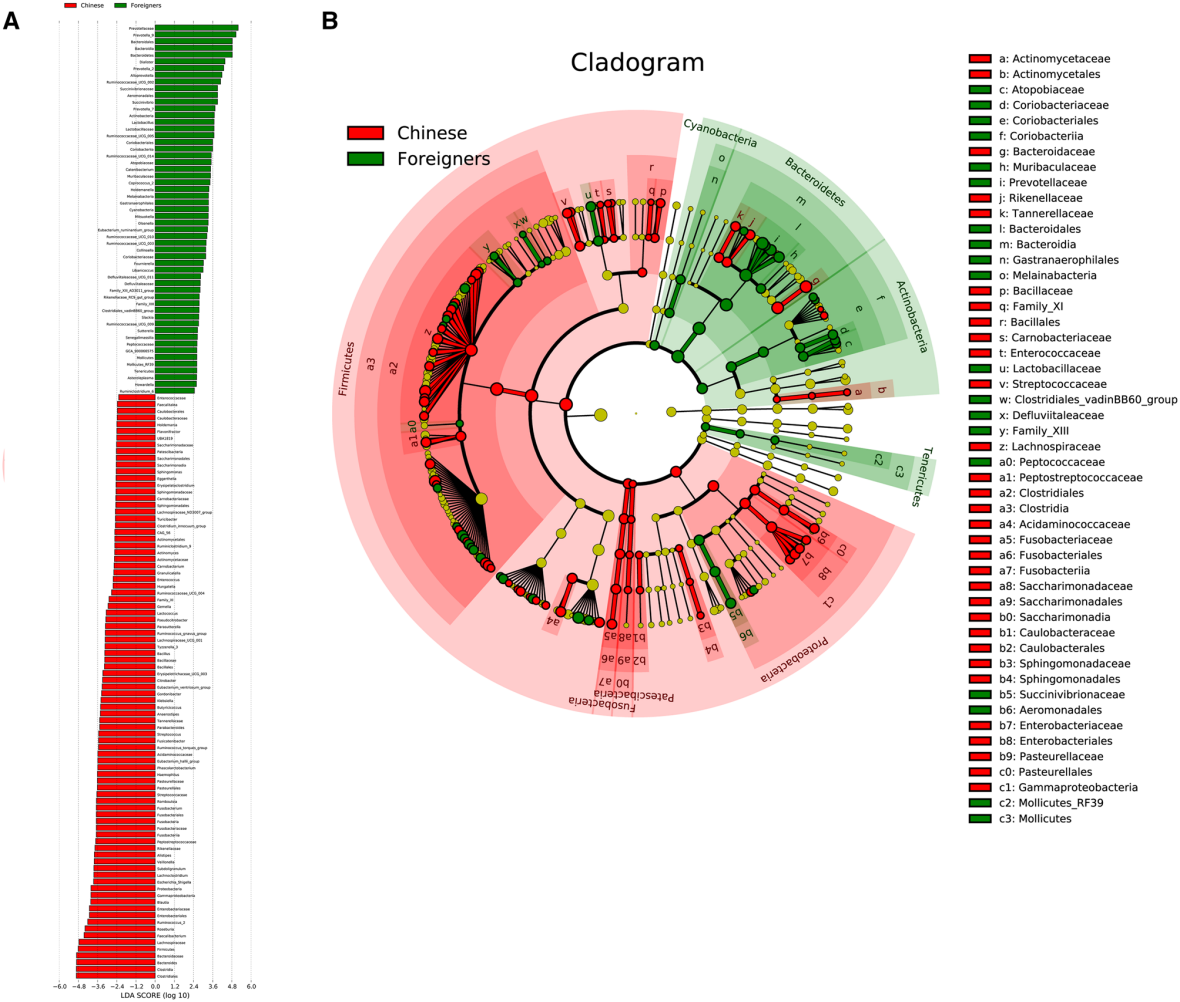
Chicken-eaters had more different genera than pork-eaters. (**A**) Biomarker taxons generated from LEfSe analysis (LDA > 2). Red bars represent samples from pork-eaters and green bars represent samples from chicken-eaters. (**B**) Cladogram obtained from LEfSe analysis with presenting various levels (phylum, class, order, family and genus) from inner to outer rings. Different color represents different groups. Different letters indicate specific gut microbial taxa from phylum to genus associated with different diets.

LEfSe analysis showed a significant difference in 142 species between the two groups (*P* < 0.05, Fig. 4). Among them, 55 species had higher abundance in chicken-eaters while 87 species had higher abundance in pork-eaters. Chicken-eaters had higher abundance of *Prevotellaceae, Prevotella 9, Bacteroidales, Dialister, Prevotella 2, Ruminococcaceae UCG 002, Lactobacillus*, and *Olsenella.* Pork-eaters had higher abundance of *Clostridiales, Bacteroides, Firmicutes, Lachnospiraceae, Faecalibacterium, Roseburia, Ruminococcus 2,* and *Blautia.*

#### Key species involved in regulating skatole, indole and SCFAs

**Figure 5:**
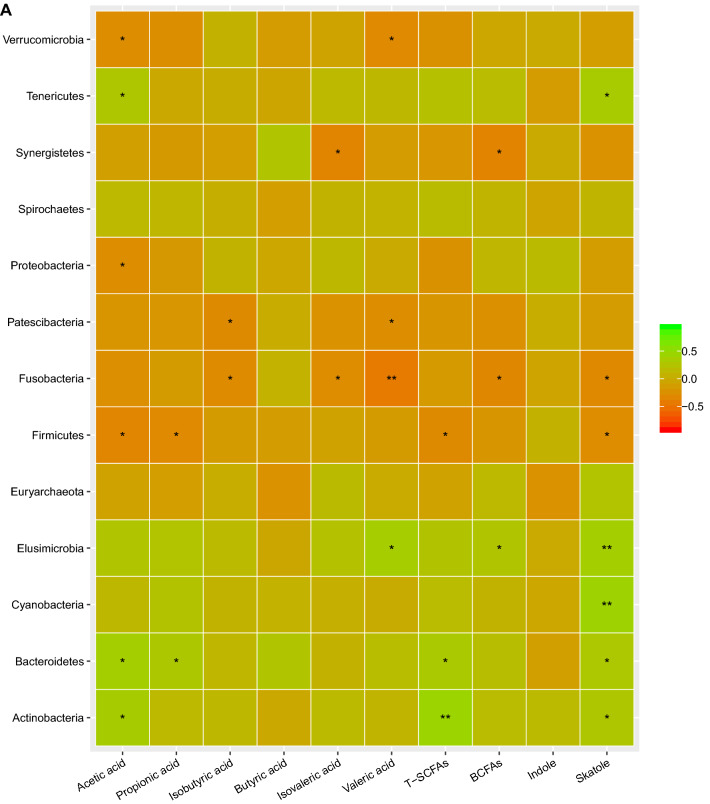

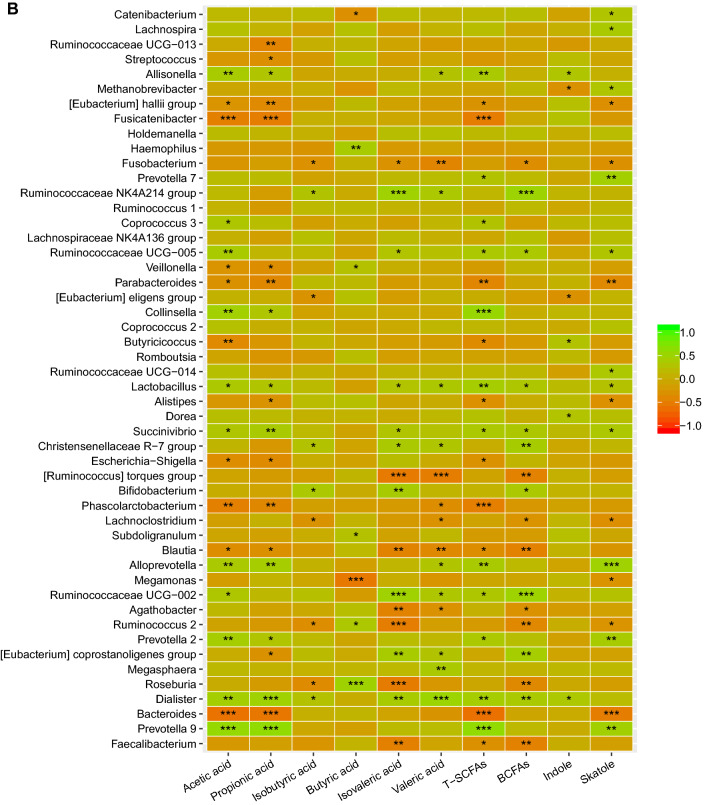
Abundance of the gut microbiota was significantly associated with levels of metabolites. (**A**) at the phyla level; (**B**) at the genus level. Green color represents positive correlation and red color represents negative correlation. Correlation was considered significant when the absolute value of Spearman’s rank correlation coefficient (Spearman’s r) was > 0.6 and statistically significant (*P* < 0.05). The asterisks indicate significant correlation, *P < 0.05; **P < 0.01; ***P < 0.001.

In order to find relationships among gut microbiota, skatole, indole and SCFAs, we performed Spearson’s correlation analysis. As shown in Fig. 5A, at the phylum level, relative abundance of *Elusimicrobia, Actinobacteria, Bacteroidetes, Cyanobacteria* and *Tenericutes* were positively correlated with skatole level. Both abundance of these bacteria and skatole level were higher in chicken-eaters than those in pork-eaters (*P* < 0.05). Relative abundance of *Firmicutes* and *Fusobacteria* were negatively correlated with skatole level. These two phyla were more abundant in pork-eaters which also had lower skatole level. Relative abundance of *Actinobacteria, Bacteroidetes* and *Tenericutes* were higher in chicken-eaters and were positively correlated with acetic acid levels. Relative abundance of *Verrucomicrobia, Proteobacteria* and *Firmicutes* was negatively correlated with acetic acid levels. Relative abundance of *Bacteroidetes* and *Firmicutes* was positively and negatively correlated with propionic acid levels, respectively. Relative abundance of *Elusimicrobia* was positively correlated with valeric acid level. Relative abundance of *Verrucomicrobia, Fusobacteria* and *Patescibacteria* was negatively correlated with valeric acid levels. Relative abundance of *Fusobacteria* and *Patescibacteria* was negatively correlated with isobutyric acid levels. *Fusobacteria* was abundant in pork-eaters and its relative abundance was negatively correlated with isovaleric acid level. Relative abundance of *Synergistetes* was negatively correlated with isovaleric acid level.

As shown in Fig. 5B, relative abundance of genera *Prevotella 9, Prevotella 7, Prevotella 2, Alloprevotella, Succinivibrio, Lactobacillu, Ruminococcaceae UCG-014, Ruminococcaceae UCG-005* and *Catenibacterium* were positively correlated with skatole level, and their abundance was higher in chicken-eaters (*P* < 0.05). Relative abundance of *Bacteroides, Ruminococcus 2, Lachnoclostridium, Alistipes, Parasutterella, Fusobacterium* and *[Eubacterium] hallii group* were negatively correlated with skatole level, and their relative abundance was higher in pork-eaters but skatole level was lower. Relative abundance of *Dialister* was positively correlated with indole and isobutyric acid levels. As mentioned above, Chicken-eaters had higher levels of indole and isobutyric acid, and higher relative abundance of *Dialister*. Relative abundance of *[Eubacterium] eligens group* and *Methanobrevibacter* were negatively correlated with indole level. Relative abundance of *Prevotella 9, Prevotella 2, Alloprevotella, Succinivibrio, Lactobacillus* and *Collinsella* were positively correlated with levels of acetic acid and propionic acid, while relative abundance of *Bacteroides, Blautia, Phascolarctobacterium, Escherichia–Shigella, Parabacteroides, Veillonella, Fusicatenibacter* and *[Eubacterium] hallii group* were negatively correlated with levels of acetic acid and propionic acid. Relative abundance of *Roseburia, Ruminococcus 2, Subdoligranulum, Veillonella,* and *Haemophilus* were negatively correlated with butyric acid level. Finally, relative abundance of *Dialister, Ruminococcaceae UCG 002* and *Lactobacillus* were positively correlated with levels of valeric acid and isovaleric acid.

## Discussion

Meat is an important source of high quality protein and essential amino acids such as tryptophan in human body^15,16^. Consumers may have different dietary habits, which has a close association with the composition of gut microbiota. Preferences for meat type differ between consumers. In a rat study with strict design, we observed that high meat protein diets caused the dysbiosis of gut microbiota and tryptophan metabolism^13^. In the present study, we for the first time investigated the gut microbiota and tryptophan metabolism in volunteers who frequently eat chicken or pork products.

Eating habits are associated with the gut microbiota composition and the profiles of gut metabolites. Dietary components, e.g., protein, fat and sugars may mediate the diet effect on host physiology and metabolism by altering the gut microbiota composition^17^. SCFAs are important metabolites of dietary fiber and protein fermented by gut microbiota^18^ and can be used as an important energy source of intestinal epithelial cells, but also affect the function of intestinal barrier^19,20^.

Meat contains high amount of tryptophan, which plays an important role in the balance between intestinal immune tolerance and the maintenance of gut microbiota. Tryptophan is absorbed in the small intestine, and the unabsorbed part reaches the cecum and colon, and is metabolized by intestinal bacteria to indole and indolic compounds, including skatole^21^. Indolic compounds can play a protective role in liver diseases including alcoholic liver disease, non-alcoholic liver disease and non-alcoholic fatty liver disease through a variety of mechanisms. Indole can promote L cells to release GLP-1 and enter the circulatory system to play an antioxidant role^22^. Small molecules produced by tryptophan metabolism, such as skatole, are sources for activating aromatic hydrocarbon receptors in different immune cells. These metabolites can regulate immunity and immune response through aromatic hydrocarbon receptors and maintain intestinal immune balance^23–26^. Skatole is an aryl hydrocarbon receptor agonist, which induces the expression of aryl hydrocarbon receptor regulatory genes, such as CYP1A2. Indole affect intestinal permeability and host immunity^27^. Skatole were shown to be pneumotoxic to humans and associated with hepatic encephalopathy and saccharo-butyric putrefaction^7–10^. Therefore, the balance of tryptophan metabolites in the gut is beneficial to maintain intestinal homeostasis and plays an important role in body health.

The present study indicated great differences in the composition of gut microbiota between chicken-eaters and pork-eaters. Pork-eaters had lower diversity of gut microbiota than chicken-eaters. *Bacteroides*, *Faecalibacterium, Roseburia*, *Dialister* and *Ruminococcus 2* were found more abundant in the feces of pork-eaters. On the other hand, *Prevotella 9*, *Dialister*, *Faecalibacterium*, *Megamonas*, and *Prevotella 2* were more abundant in the feces of chicken-eaters. Such a difference could be attributed to dietary structure that is more complex than a strictly controlled diet in our previous rat study^13^. In that study, chicken and pork protein diets increased the abundance of *Lactobacillus*, *the Family XIII AD3011 group*, and *Desulfovibrio* in rat large intestine relating to the production of skatole and indole^13^. A previous comparative study revealed that differences in dietary habits led to significant differences in the composition of gut microbiota, in which African children who eat foods rich in plant fiber had higher levels of *Prevotella* and *Xylanibacte,* as well as SCFAs and *Enterobacter*^28^. A study comparing the gut microbiota of Japanese and Indian with different dietary preferences showed that the abundance of *Bacteroides* was higher in Japanese, while the abundance of *Prevotella* was higher in Indian^29^. The altered gut microbiota may affect tryptophan metabolism^30,31^. Chicken-eaters had higher fecal skatole and indole, and higher abundance of *Prevotella 2* and *Prevotella 9.* Chicken-eaters exhibited significantly higher contents of T-SCFAs. This could be because more diet-derived substances went into the large intestine for the fermentation of gut microbiota.

In summary, the effect of meat diet mode was explored on human gut microbiota composition and tryptophan metabolites, i.e., skatole and indole. The dietary mode had a significant effect on the gut microbiota composition and the levels of skatole and indole. Chicken-eaters had higher relative abundance of *Prevotella 9, Prevotella 2, Dialister, Faecalibacterium,* and *Megamonas,* and higher levels of SCFAs and tryptophan metabolites. In particular, relative abundance of *Prevotella 2* and *Prevotella 9* was positively correlated with levels of fecal skatole, indole and SCFAs. However, pork-eaters had higher relative abundance of *Bacteroides, Dialister, Faecalibacterium, Roseburia,* and *Ruminococcus 2*. The results give an indication that long-term intake of chicken diet may increase the risk of skatole- and indole-induced diseases by altering gut microbiota.

## Methods

### Volunteers and sample collection

Male volunteers of 18 and 30 years old were recruited to carry out a dietary questionnaire (supplementary file 1) to investigate the dietary habits and medical history, and to collect their living habitual and long-term dietary information. In the study, 40 eligible healthy volunteers who did not consume any antibiotics for 3 months participated. Informed consent was issued to volunteers who met the sampling standards, and the experiment was conducted after obtaining written consent. According to the survey results of dietary structure, volunteers were divided into two groups, namely the chicken-eaters (n = 20) and pork-eaters (n = 20). Chicken-eaters prefer chicken as the main meat dishes, whereas pork-eaters prefer pork as the main meat dishes.

The sterilized feces collectors were distributed to volunteers. Fecal samples were collected at morning and frozen in liquid nitrogen, and stored at − 80 °C for further analyses. This study followed the Declaration of Helsinki. The research protocol was approved by the Ethics Committee of Nanjing Agricultural University (Nanjing) and registered in the World Health Organization clinical trial registration platform (WHO ICTRP) first-level registration institutions (registration number: ChiCTR1800015339). All the procedures were conducted in strict accordance with the approved guidelines.

### Skatole and indole quantification

Ultraperformance liquid chromatography (UPLC, Agilent) was used to quantify skatole and indole in feces as we previously described^13^. Briefly, skatole and indole were extracted by mixing fecal samples (50 mg) with 500 μL of methanol and then centrifuging for 10 min at 3000*g*. The supernatants (4 µL) were separated in a C18 column (1.8 μm, 50 × 2.1 mm, Agilent, California) and analytes were detected by a UPLC with a fluorescence detector (G1321B, λex = 280 nm, λem = 360 nm). The mobile phase A was ultra-pure water, and mobile phase B was acetonitrile. The elution conditions were as follows: 0–6 min, 90% A; 6–10 min, 60% A; and 10–12 min, 40% A. By comparing with retention time of standards, contents of skatole and indole in samples were qualitatively determined, and the concentration was calculated by the standard curve using an external standard method.

### SCFAs analysis

Fecal samples (40 mg) were diluted in 200 μL ddH_2_O and centrifuged for 10 min at 12,000*g*. An internal standard (crotonic acid) was prepared by mixing 34 μL of crotonic acid with 170 μL of the supernatant. Centrifugation was repeated once. The supernatant (1 mL) was injected into a gas chromatograph (GC-2010 Plus Shimadzu, Japan) equipped with a HP-INNOWax capillary column (30 m × 0.25 mm × 0.25 μm, Agilent Technologies, CA). The chromatographic parameters were performed with a capillary pipette column and a hydrogen ion flame detector. The temperatures for column, vaporization and detection were set 130 °C, 180 °C, and 180 °C, respectively. The carrier gas was nitrogen with a pressure of 60 kPa. The oxygen pressure was set at 50 kPa. The sensitivity (gear) was 10^–1^, and the attenuation was 3.0.

### 16S rRNA gene sequencing and data analysis

Total genome DNA was extracted from fecal samples with an Omega Bio-tek DNA extraction kit (Norcross, GA). DNA samples were amplified with universal primers F341 (5ʹ-CCTAYGGGRBGCASCAG-3ʹ) and R806 (5ʹ -GGACTACNNGGGTATCTAAT-3ʹ). The pair-end library was constructed and the amplicons were sequenced on an Illumina MiSeq platform. Sequencing data were analyzed as previously described^13^, including bacterial diversity (Shannon and Simpson) and community richness (Chao) of operation taxonomy units (OTUs). The UniFrac for principal coordinate analysis (PCoA) was done to estimate the beta diversity of gut microbiota. Linear discriminant analysis effect size (LEfSe) was performed to identify the bacterial biomarkers between chicken-eaters and pork-eaters. The Spearman’s correlation coefficients between gut microbiota and skatole, indole or SCFAs (r > 0.6, *P* < 0.05) were visualized on the R program (version 3.2.4, https://www.r-project.org).

### Statistical analysis

The differences between the two groups in measured variables were evaluated by Student’s t-test. The significance level was set as 0.05. The SAS program was applied for the analyses (SAS, version 8.0.1, Cary, NC).

## Supplementary Information

Supplementary Information.
