# Supplementary material for: Chicken-eaters and pork-eaters have different gut microbiota and tryptophan metabolites
Source: Sci Rep. 2021 Jun 7;11:11934. doi: 10.1038/s41598-021-91429-3 (PMC8184825; doi:10.1038/s41598-021-91429-3)
Supplement: Supplementary file 1 — Supplementary Information. [file 41598_2021_91429_MOESM1_ESM.pdf]

Chicken-eaters and pork-eaters have different gut microbiota and tryptophan metabolites

Jie Shi[a], Di Zhao[a], Fan Zhao[a], Chong Wang[a], Galia Zamaratskaia[b, c], Chunbao Li[a, *]

[a] Key Laboratory of Meat Processing and Quality Control, MOE; Key Laboratory of Meat Processing, MARA; Jiangsu Synergistic Innovation Center of Meat Processing and Quality Control, College of Food Science and Technology, Nanjing Agricultural University, 210095, Nanjing, P.R. China;

[b] Department of Molecular Sciences, Swedish University of Agricultural Sciences, 75007, Uppsala, Sweden;

[c] University of South Bohemia in Ceske Budejovice, Faculty of Fisheries and Protection of Waters, South Bohemian Research Center of Aquaculture and Biodiversity of Hydrocenoses, Zatisi 728/II, 389 25 Vodnany, Czech Republic

***Corresponding author:** Dr. Chunbao Li

Address: College of Food Science and Technology, Nanjing Agricultural University, 210095, Nanjing, P.R. China

Tel: 0086 25 84395679; E-mail: chunbao.li@njau.edu.cn

Running title: meat diet, gut microbiota and tryptophan metabolism

Keywords: meat; gut microbiota; tryptophan metabolism; skatole; indole

Diet questionnaire

Section I. Personal information

Name:	Sex:	Age:
College:	Ethnicity:	Height: cm
Weight: kg	Blood type:	Residence:
Tel:	QQ:	WeChat:
Religious beliefs:	Country:	

Section 2. Living habits:

1. Which meat do you prefer to eat? (please ticket the items you prefer)

☐ Pork ☐ Chicken ☐ Beef ☐ Lamb ☐ Duck ☐ Fish ☐Others

2 . What about frequencies to eat meat per week? (please ticket the items you prefer)

☐ 1-2 times ☐ 3-5 times ☐ more than 5 times

3. Do you have any strenuous exercise (running, basketball, soccer, etc.)?

☐ Yes ☐ No

4. What about frequencies to exercise per week? (please ticket the items you prefer)

☐ 1-2 times ☐ 3-5 times 3) More than 5 times

5. How long time do you exercise each time? ( ) hours

6. Do you smoke? ☐ Yes ☐ No

7. Do you drink often? ☐ Yes ☐ No

8. How often do you drink per week? (please ticket the items you prefer)

☐ Do not drink ☐1-2 times ☐ 3-5 times ☐More than 5 times

9. When do you have your meals? (breakfast: lunch: dinner:)

10. Whether you have a long-term consumption of yogurt and other fermented dairy products? (please ticket the items you prefer)

☐1-2 times a week ☐ 3-5 times a week ☐ More than 5 times a week

11. Do you live in school? (please ticket the items you prefer)

☐Yes ☐ No

12. Which is your main food? (please ticket the items you prefer)

☐ rice ☐ pasta (bread, bread) ☐ whole grains ☐ potato ☐ meat ☐ others (please specify)

13. Which vegetables do you prefer?

☐ leafy vegetables ☐rhizomes ☐melon ☐ fresh beans ☐ fungi

14. Do you regularly consume seasonal fruits?

☐ apple ☐orange ☐banana ☐pear ☐jujube ☐others (please specify)

15. How much water do you drink (250ml a cup)?

☐ 1 cup ☐ 2-3 cups ☐ 4-5 cups ☐ 6 cups or more

16. Do you have breakfast?

☐ Yes ☐ No

17. What does your breakfast consist of?

☐ bread, milk ☐ buns, porridge ☐ hamburger, fruit juice ☐ fried dough sticks, soy milk

18. How much do you eat in breakfast?

19. What does your lunch consists of? How much do you eat in lunch?

20. What does your supper consists of? How much do you eat in supper?

21. Do you eat spicy foods?

☐ yes ☐ no

24. Do you have insomnia usually?

☐ yes ☐ no

25. How long time do you sleep every day? () hours

Section 3. Health information

1. What about your overall health status?

☐ Good ☐ Fair ☐ Poor

2. Do you have a history of intestinal disease? ☐Yes ☐ No

3. Do you have constipation? ☐Yes ☐No

4. Do you have a history of allergy? ☐Yes ☐No

5. Do you have a history of chronic diseases? ☐Yes ☐ No

6. Have you taken antibiotics or probiotics products in recent three months? ☐ Yes ☐ No
